# Association of Increased Plasma Interleukin-6 and TNF-α Levels in Donors with the Complication Rates in Liver Transplant Recipients

**Published:** 2013-02-01

**Authors:** N. Azarpira, S. Nikeghbalian, K. Kazemi, B. Geramizadeh, Z. Malekpour, S. A. Malek-Hosseini

**Affiliations:** 1 *Shiraz Transplant Research Center, *; 2 *Organ Transplantation Center, Shiraz University of Medical Sciences, Shiraz, Iran*

**Keywords:** Transplantation, complications, Cytokine, Interleukin, Tumor necrosis factor alpha

## Abstract

Background: Solid organ transplantation is the only definitive treatment available for patients with end-stage organ failure. Organs procured from brain-death donors are the main source of transplants. Following brain death, a burst of inflammatory reaction develops; it is characterized by increased plasma levels of cytokines. This inflammatory reaction has been associated with increased early allograft dysfunction.

Objective: In this study, we test if the increased inflammatory response in brain-death donors is associated with more recipient complications.

Methods: We prospectively recruited 38 consecutive brain-death donors admitted to the intensive care units (ICUs) of Shiraz University of Medical Sciences. Following the declaration of brain death, the demographics data on donor and recipient characteristics and cause of brain death were recorded. The post-liver transplant complications in recipients were stratified according to the Clavien classification. Plasma levels of cytokines IL-6, IL-2, and TNF-α were measured using enzyme linked immunosorbent assay (ELISA) kits, in all donors before organ procurement.

Results: The mean (range) age of donors was 44 (16–74) years. Trauma due to car accident was the most common cause of brain death (79%). The post-liver transplant complications occurred in 19 (50%) recipients. The mean±SD plasma TNF-α concentration was significantly (p<0.001) higher in recipients with grade 1-3 post-transplant complications (68.33±27.74 pg/mL) than those without complication (22.09±4.14 pg/mL). Recipients with complications had also a significantly (p=0.001) higher mean±SD donor plasma concentration of IL-6 (1009±375.5 pg/mL) compared to those without complications (779±202 pg/mL). No significant differences was observed between the two groups in respect to IL-2 concentration (0.295±0.333 *vs* 0.285±0.342 U/mL, p=0.207). Six recipients died of complications (grade5), in whom no correlation could be found with donor plasma cytokine concentrations.

Conclusion: Higher plasma concentrations of IL-6 and TNF-α in donors before organ procurement, are associated with more post-operative complications in liver transplant recipients.

## INTRODUCTION

Solid organ transplantation is the only definitive treatment available for patients with end-stage organ failure. There is a large disparity between the transplant waiting list candidates and the number of available organs in the world. Factors such as donor age, comorbidities and infection are among important reasons that many organs are found not suitable for transplantation [1-3]. Organs derived from brain-death donors are the main source of transplants.

Following brain death, a burst of inflammatory reaction develops; it is characterized by increased plasma levels of cytokines [4, 5]; elevated plasma level of interleukin-6 (IL-6) occurred after neuronal injury [6, 7]. This inflammatory reaction has been associated with increased early allograft dysfunction [8]. In heart transplantation, high tissue and plasma levels of interleukin-1 (IL-1), IL-6 and tumor necrosis factor alpha (TNF-α) were associated with worse donor heart function [9] and post-transplantation graft outcome [2, 9]. C-reactive protein (CRP) and procalcitonin (PCT) were also markers of inflammation; elevated levels of CRP and PCT were observed after brain death and associated with adverse adverse outcomes in heart transplant recipients [2]. Diminished circulating thyroid hormone levels, tri-iodothyronine (T3), was developed in donors; the hormone is commonly administered to donors as a putative optimizing agent. Administration of steroids also modulated the inflammatory environment of donor organs by stabilization of cell membrane, reduction of HLA antigen upregulation, and inhibition of cytokine elaboration [2]. 

Available studies reveled an inflammatory response in the liver following brain death [10, 11]. Experimental studies confirmed lower graft survival for the heart, lung and kidneys procured from brain-death donors compared with living donors [11-13].

The risk of kidney and liver graft dysfunction is higher in grafts taken from brain-death donors than in grafts procured from living donors [14, 15]. We conducted this study to determine if higher inflammatory response in donors is associated with more complication rates in liver transplant recipients.

## PATIENTS AND METHODS

We prospectively recruited 38 consecutive brain-death donors admitted to the intensive care units (ICUs) of Shiraz University of Medical Sciences between April and November 2010. The study was approved by the Ethics Committee of Shiraz University of Medical Sciences. The consent for organ donation was obtained from first degree relatives.

Donors with leucopenia, history of recent chemotherapy, or no heart beating were excluded from the study. Demographics and etiology of brain death were recorded. Decisions regarding organ procurement and transplantation were made by an expert surgical team. Organs might be discarded after procurement due to organ dysfunction as assessed by biopsy, other physiological variables, or visual quality of the organs as confirmed by the transplant surgeons. The post-liver transplant complications in recipients were classified according to Clavien grading system [16]. Demographic data of recipients with post-transplant complications were also recorded.

Cytokine measurement 

Plasma cytokines IL-6, IL-2, and TNF-α were measured in all donors before organ procurement. Samples were drawn from pre-existing arterial cannula; plasma was separated, divided into three separate 1.5-mL tubes and stored frozen at -80 °C until they were assayed. Each sample was used for one assay to avoid multiple freezing/thawing cycles.

The IL-6, IL-2, and TNF-α concentrations were measured with enzyme linked immunosorbent assay (ELISA) commercial kits (DRG, Germany), using an automated immunoassay analyzer (Stat Fax, Los Angeles, CA, USA). The normal reference range for each cytokine was 0–50 pg/mL for IL-6; 0–0.1 U/mL for IL-2; and 4.6–12.4 pg/mL for TNF-α.

Statistical analysis 

Data are expressed as means±SD. Analyses were performed with SPSS^®^ ver 15.0 for Windows^®^ (SSPS Inc, Chicago, Ill, USA). The level of plasma cytokines were compared between recipients with grade 1-3 and grade 5 complication by Mann-Whitney U test. A p value <0.05 was considered statistically significant.

## RESULTS

The mean±SD (range) age of the donors was 44±10.2 (16–74) years. Trauma due to car accident was the most common cause (79%) of brain death (Table 1). Donors were not treated with corticosteroids following brain death.

**Table 1:** Demographic and clinical characteristics of brain-death donors (n=38)

**Parameter**	**Value**
Age (mean±SD) (yrs)	22±15.5
Gender (%)	
Male Female	32 (84%) 6 (16%)
Mechanism of brain death (%)	
Subarachnoid hemorrhage Intracerebral hemorrhage Closed head injury due to car accident Gun shot injury	2 (5%) 5 (13%) 30 (79%) 1 (3%)
Time to organ procurement after brain death (mean±SD) (hrs)	12±10.1

The mean±SD (range) age of recipients was 22±15.5 (2–55) years. Demographic and clinical characteristics of organ recipients are presented in Table 2.

**Table 2:** Demographic and clinical characteristics of liver transplant recipients (n=38)

**Cause of cirrhosis**	**n (%)**	**Male/ Female**
Hepatitis B virus	5 (14)	3/2
Autoimmune hepatitis	4 (11%)	2/2
Primary sclerosing cholangitis	3 (9%)	2/1
Hepatitis C virus	2 (5%)	0/2
Hemochromatosis	2 (5%)	2/0
Primary biliary cirrhosis	2 (5%)	1/1
Wilson’s disease	2 (5%)	1/1
Tyrosinemia	1 (3%)	1/0
Neonatal hepatitis	1 (3%)	1/0
Hypercholestrolemia	1 (3%)	1/0
Budd Chiari	1 (3%)	0/1
Progressive familial intrahepatic cholestasis	1 (3%)	1/0
Tyrosinemia	1 (3%)	0/1
Cryptogenic cirrhosis	12 (33%)	8/4

The complications in 19 (50%) recipients were classified according to Clavien grading system (Table 3). Grade 1 complications were recorded in 2.6%; grade 2 in 10%; grade 3a in 8%; grade 3b in 13.5%; and grade 5 in 15.7% of recipients—there was no grade 4 complication. One common complication was portal vein thrombosis requiring relaparatomy (grade 3b).

**Table 3:** Complications of 38 liver transplant recipients classified according to Clavien system

**Grades**	**n**	**Complication**	**n**
**Grade 1 (n=1, 3%)**	1	Chicken pox infection	1
**Grade 2 (n= 4, 10%)**	1 2 1	Cytomegalovirus infection requiring pharmacological treatment Bleeding needing blood transfusion Pneumonia requiring antibiotics	1 2 1
**Grade 3a (n= 3, 8%)**	1 1 1	Bile leakage needing ERCP or surgical intervention Non-Hodgkin’s lymphoma requiring pharmacological treatment Pneumonia due to H1N1 that needs medical treatment	1 1 1
**Grade 3b (n=5, 14%)**	4 1	Portal vein thrombosis requiring relaparotomy Bowel perforation requiring relaparotomy	4 1
**Grade 4a, 4b**	0	—	0
**Grade (n=6, 16%)**	6	Death	6

ERCP: Endoscopic retrograde cholangiopancre- atography

The mean±SD donor plasma cytokine concentrations were 1040.20±1200.47 pg/mL for IL6, 0.312±0.41 U/mL for IL2, and 37.22±59.40 pg/mL for TNF-α. TNF-α concentration was significantly (p<0.001) higher in recipients with grade 1-3 complications (68.33±27.74 pg/mL) than those without complications (22.09±4.14 pg/mL). Recipients with grade 1-3 complications had a significantly (p=0.001) higher donor plasma concentration of IL-6 (1009±375.5 pg/mL) than recipients without complications (779±202 pg/mL). No significant difference was observed between mean concentrations of IL-2 in the two groups (0.295±0.333 *vs* 0.285±0.342, p=0.207).

There were six deaths (grade 5 complication) in recipients which occurred within the first two months of transplantation. No correlation could be found between donor plasma concentration of IL-2, IL-6, and TNF-α and recipient’s death. There was also no statistically significant difference between complication rates in recipients who received organs from donors aged <50 *vs* those received organs from donors age >50 years. There was also no statistically significant difference between the underlying disease in recipients and post-transplant complications.

## DISCUSSION

“Brain death” is believed to be an important antigen-independent risk factor for harming organs before transplantation [1, 4]. Donor organs can be damaged through several mechanisms. “Catecholamine storm” produced from ischemic brain stem, results in a dramatic hypertensive crisis and peripheral vasoconstriction which lead to organ ischemia [1, 4]. In patients with brain injury, jugular IL-6 levels were higher than its arterial levels, suggesting that IL-6 had originated from the brain [17, 18]. Elevated levels of circulating cytokines, especially IL-6, would induce a local inflammatory response in the recipients’ organs [18-20]. An inverse correlation between plasma levels of IL-6 and kidney graft survival was reported [1, 21, 22]. Elevated levels of IL-8 and IL-10 was also detected in the plasma of brain-death donors [1, 23]. An important family of protein kinases involved in signal transduction is the MAP kinases. This pathway is regarded as key players in the inflammatory response in the kidney after brain death. MAP kinases are activated by a variety of stimuli including cytokines (IL-6), growth factors, hormones, pathological stressors like ischemia/reperfusion, ultraviolet irradiation and neuronal injury [24, 25].

The MAP kinase-mediated inflammatory response stimulates production and activation of chemokines, selectins and adhesion molecules and leads to subsequent leukocyte infiltration in the deceased organ. The pre-existing organ injury is then aggravated by cold ischemia (organ preservation) and during the transplantation operation (ischemia/reperfusion injury) [1, 25].

We found that higher donor plasma concentrations of IL-6 and TNF-α were associated with higher post-transplant complications. Our finding is consistent with other studies showing that inflammatory response after brain death is associated with lower organ viability and contributes to early allograft dysfunction in organ recipients [2, 3].

Recipients with deceased donor kidneys have a significantly increased risk of delayed graft function and inferior long-term graft survival compared with those received transplants from living donors [1-3].

Venkateswaran, *et al*., found that high TNF- α level, but not IL-6, was associated with donor cardiac dysfunction [2]. Activation of IL-6/IL-6 receptors in donor hearts has been associated with early cardiac allograft dysfunction after cardiac transplantation in the absence of cellular rejection [8].

Brain death also induces an inflammatory response in the liver [9, 10, 15]. In a study performed by Jassem, *et al*. [10], the biopsies were taken after cold ischemia. The pathological finding of liver was significantly different between brain-death and living donors.

Livers procured from the brain-death donors were associated with a high rate of rejection and primary nonfunction in one study [15]. No association was found between plasma cytokine concentration and two-month graft survival in liver transplant recipients [15].

Murugan, *et al*., measured TNF, IL-6, and IL-10 in donors before organ procurement for transplantation [3], and found that all cytokines were increased following brain death. However, only high plasma IL-6 concentrations in donors before organ procurement was significantly associated with lower hospital-free survival in recipients [3].

Overall, “cytokine storm” in the human donor was associated with decreased organ yield and reduced hospital-free survival in their recipients [2].

In conclusion, elevated plasma IL-6 and TNF-α concentrations before organ procurement are associated with more complication rates in recipients. Brain-death-related systemic inflammatory response in the donor adversely affects recipient outcome. These data provide a rationale to study the immune modulation therapies in donors to improve organ function and recipient outcome. One of the important limitations of our study was lack of age- and sex-matched controls for the level of cytokines. This warrants conduction of controlled studies.
